# Correction: Ma et al. The Effect of Virtual Reality Technology in Table Tennis Teaching: A Multi-Center Controlled Study. *Sensors* 2024, *24*, 7041

**DOI:** 10.3390/s25082561

**Published:** 2025-04-18

**Authors:** Tingyu Ma, Wenhao Du, Qiufen Zhang

**Affiliations:** 1 School of Physical Education and Sport, Henan University, Kaifeng 475004, China; 17539120574@163.com; 2 School of Economics and Management of Shanghai University of Sports, Shanghai 200438, China; wenhao@henu.edu.cn

In the original publication [1], the reference “Oagaz, H.; Schoun, B.; Choi, M.H. Performance Improvement and Skill Transfer in Table Tennis Through Training in Virtual Reality. *IEEE Trans. Vis. Comput. Graph.* **2021**, *27*, 4332–4343. https://doi.org/10.1109/TVCG.2021.3086403” was not cited. The citation has now been inserted in the caption of Figure 1 in Section 1 and reads as follows:

**Figure 1.** Virtual reality table tennis training environment. Reprinted with permission from *IEEE Transactions on Visualization and Computer Graphics*, Copyright 2022, IEEE [7].

The following new Reference [7] has been added: 

7. Oagaz, H.; Schoun, B.; Choi, M.H. Performance Improvement and Skill Transfer in Table Tennis Through Training in Virtual Reality. *IEEE Trans. Vis. Comput. Graph.* **2021**, *27*, 4332–4343. https://doi.org/10.1109/TVCG.2021.3086403.

To accommodate this correction, the order of some references was adjusted accordingly. The authors state that the scientific conclusions are unaffected. These corrections were approved by the Academic Editor. The original publication has also been updated.
